# Environmentally friendly synthesis of CeO_2_ nanoparticles for the catalytic oxidation of benzyl alcohol to benzaldehyde and selective detection of nitrite

**DOI:** 10.1038/srep46372

**Published:** 2017-04-13

**Authors:** P. Tamizhdurai, Subramanian Sakthinathan, Shen-Ming Chen, K. Shanthi, S. Sivasanker, P. Sangeetha

**Affiliations:** 1 Department of Chemistry, Anna University, Chennai 600025, India; 2 National Centre for Catalysis Research, Indian Institute of Technology, Chennai, India; 3 Electroanalysis and Bioelectrochemistry Lab, Department of Chemical Engineering and Biotechnology, National Taipei University of Technology, No. 1, Section 3, Chung-Hsiao East Road, Taipei 106, Taiwan (ROC); 4 Department of Chemistry, School of Advanced Sciences, VIT University, Vellore, India.

## Abstract

Cerium oxide nanoparticles (CeO_2_ NPs) are favorable in nanotechnology based on some remarkable properties. In this study, the crystalline CeO_2_ NPs are successfully prepared by an efficient microwave combustion (MCM) and conventional route sol-gel (CRSGM) methods. The structural morphology of the as-prepared CeO_2_ NPs was investigated by various spectroscopic and analytical techniques. Moreover, the XRD pattern confirmed the formation of CeO_2_ NPs as a face centered cubic structure. The magnetometer studies indicated the low saturation magnetization (23.96 emu/g) of CeO_2_ NPs for weak paramagnetic and high saturation magnetization (32.13 emu/g) of CeO_2_ NPs for super paramagnetic. After that, the oxidation effect of benzyl alcohol was investigated which reveals good conversion and selectivity. Besides, the CeO_2_ NPs modified glassy carbon electrode (GCE) used for the detection of nitrite with linear concentration range (0.02–1200 μM), low limit of detection (0.21 μM) and higher sensitivity (1.7238 μAμM^−1^ cm^−2^). However, the CeO_2_ NPs modified electrode has the fast response, high sensitivity and good selectivity. In addition, the fabricated electrode is applied for the determination of nitrite in various water samples. Eventually, the CeO_2_ NPs can be regarded as an effective way to enhance the catalytic activity towards the benzyl alcohol and nitrite.

Over the last decade, metal and metal oxide nanoparticles are an emerging field of nanoscience and technology. The size and shape of nanomaterials significantly plays a vital role in physical, chemical, electrical and optical properties^1^. In recent years, cerium oxide nanoparticles (CeO_2_ NPs) have been widely used in catalysis, energy storage, optical sensor and biomedicine application due to the unique physical and chemical properties^2^,^3^. Moreover, the controlled synthesis of CeO_2_ based nanomaterials have more attention for redox reactivity and oxygen transport properties compared with bulk CeO_2_ raw materials^4^. Besides, the most remarkable activity of CeO_2_ obtained from the surface of oxygen releasing electrons, which may reduce the ceric ions (Ce^4+^) into cerous ions (Ce^3+^). Hence, the redox properties (Ce^4+^/Ce^3+^) and oxygen exchange are dramatically increased due to its nanoscale dimension and activity^5^. In addition, several techniques have been applied for controlled synthesize of CeO_2_ NPs such as hydrothermal, flame spray pyrolysis, microwave assisted combustion, sonochemical and co-precipitation methods^6^,^7^,^8^,^9^,^10^. Eventhough, the microwave assisted combustion method (MCM) has emerged as a novel method for producing many nanomaterials in causes of heat is generated within material internally, growing rapidly, increasing reaction rate and shortened reaction time. However, in these method involved environmental toxic chemicals as a reducing agents therefore, we need to develop an effective and environmental friendly method for the nanomaterials synthesis^11^,^12^,^13^,^14^.

The conventional route sol-gel method (CRSGM) is a useful and attractive technique for the preparation of metal oxides nanoparticles. Nowadays, plant extracts have been used as a reducing and capping agent for the synthesis of metal oxide nanoparticles, which is act as a fuel and plays a coordinating agent^15^. Especially, the Aloe vera plant contains high water content (97.5–99.5%), fat-soluble vitamins, minerals, enzymes and polysaccharides^16^. These plant extract used as a bio-reducing agent for the preparation of metal oxide nanoparticles. It is also noticed as a cheap precursor and provides high-yield of CeO_2_ NPs with well crystalline structure^17^,^18^. Moreover, aldehydes and ketones are important raw materials for the synthesis of many fine chemicals such as, dyes, medicines and perfumes. The most important conversion of carbonyl compounds is the selective oxidation of benzyl alcohol to benzaldehyde. However, some suitable catalyst always plays an important role in these conversion reactions. Especially, CeO_2_ NPs used as a catalyst and plays an important role due to the extraordinary catalytic activity^19^.

Nitrite is widely used in food, beverage, fertilizer manufacturing and corrosion inhibitor. The permissible range of nitrite in water is 1 mg/L and the excess of nitrite in water can causes several problems to human such as, blue baby syndrome and shortness of breath^20^,^21^,^22^. Hence, the sensitive and selective detection of nitrite in food, water and environmental samples is more important^23^. Therefore, several analytical techniques have been developed for the detection of nitrite, such as chemiluminescence, chromatography, spectrometry and capillary electrophoresis^24^,^25^,^26^. However, it must be note that, these methods involved in time consuming, expensive and tedious protocol. In contrary, the electrochemical methods used for the detection of nitrite due to the simple procedure, rapid response and high sensitive methods than that of the other aforementioned analytical methods^27^,^28^.

In addition, the electrochemical detection of nitrite at unmodified conventional electrode is poor in the presence of other oxidizing agents. Therefore, the chemically modified glassy carbon electrode (GCE) has mostly been used for the detection of nitrite at low overpotential with higher detection sensitivity. Hence, the CeO_2_ NPs modified electrode exhibited higher catalytic activity towards the detection of nitrite due to it’s unique catalytic and electron transfer properties^29^. To the best of our knowledge, there is no report for the conventional route sol-gel (CRSGM) method prepared CeO_2_ NPs used as a catalyst for benzyl alcohol to benzaldehyde reaction and nitrite sensor. As illustrated in Fig. 1, the CeO_2_ NPs exhibits the higher catalytic activity towards benzyl alcohol oxidation and detection of nitrite. Furthermore, the morphology and catalytic performance of CRSGM method prepared CeO_2_ NPs was compared with the MCM method prepared CeO_2_ NPs.

## Results and Discussion

### X-Ray diffraction studies

**Figure 2:**
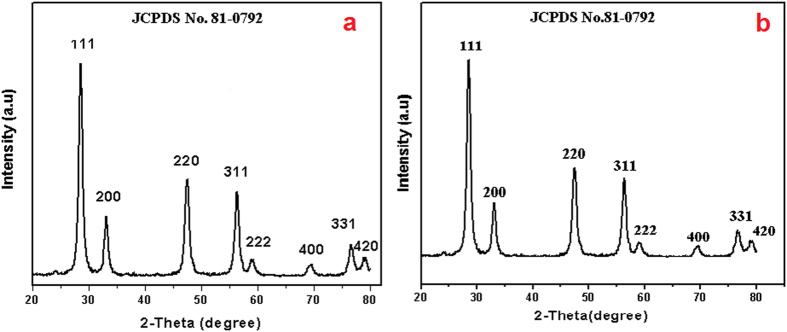
XRD pattern of (**a**) CeO_2_ NPs prepared by the MCM and (**b**) CeO_2_ NPs prepared by the CRSGM.

The structural phases of the CeO_2_ NPs were determined by X-ray diffraction studies. Figure 2a and b shows the XRD pattern of MCM prepared CeO_2_ NPs and CRSGM prepared CeO_2_ NPs, respectively. Moreover, the diffraction peaks observed at 2θ = 28.66, 33.03, 47.56, 56.39, 59.05, 69.34, 76.61 and 79.27 are corresponding to (111), (200), (220), (311), (222), (400), (331), and (420) planes. The obtained reflections corresponding to the face-centered cubic phase with the lattice parameter of a = b = c = 0.5412 nm. These corresponding planes are associated with the d-spacing values of 3.12, 2.70, 1.91, 1.63, 1.56, 1.35, 1.24 and 1.21 Å. Which can be readily assigned to a cubic phase of CeO_2_ NPs (JCPDS file No: 81–0792). Moreover, the calculated value of ‘a’ is 5.426 Å for CeO_2_ NPs and the unit cell volume is calculated by V = a^3^.

In addition, the unit cell volume is found to be 157.790 Å for the CeO_2_ NPs and there is no additional peak in the XRD patterns which revealed that the high purity of CeO_2_ NPs. Besides, the strong and sharp diffraction peaks indicated the good crystallinity of the CeO_2_ NPs. The average crystallite size of CeO_2_ NPs were calculated by using Debye Scherrer formula^29^.


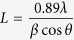


Where, L is the average crystallite size (Å), λ is the X-ray wavelength (0.154 nm), θ is the diffraction angle and β is the full-width of the observed peak. The average crystallite size of the MCM (**sample-*****a***) and CRSGM (**sample-*****b***) methods prepared CeO_2_ NPs was found to be 23.83 nm to 18.23 nm, respectively. The obtained crystallographic parameter of CeO_2_ NPs are indicated in Table S1. The obtained crystallographic properties of the CeO_2_ NPs are in good agreement with the previous report^30^.

### High resolution scanning electron microscopy (HR-SEM) and transmission electron microscopy (HR-TEM) studies

**Figure 3:**
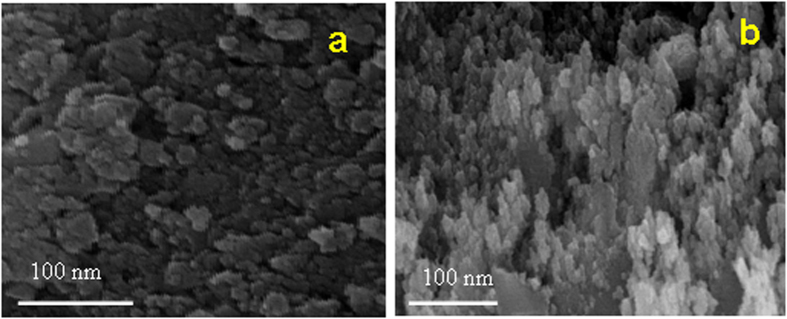
SEM images of (**a**) CeO_2_ NPs prepared by the MCM and (**b**) CeO_2_ NPs prepared by CRGSM.

**Figure 4:**
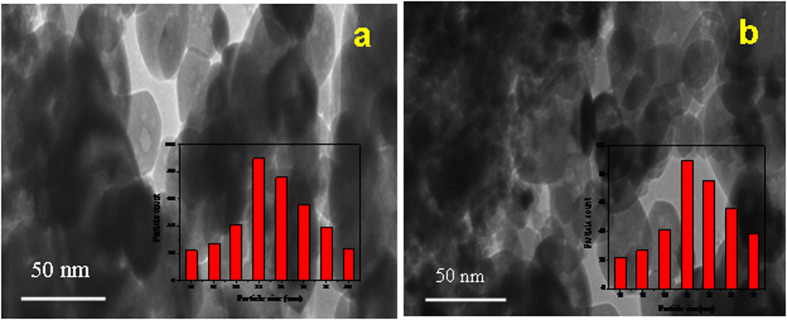
HR-TEM images of (**a**) CeO_2_ NPs prepared by the MCM and (**b**) CeO_2_ NPs prepare by the CRSGM.

Surface morphologies of CeO_2_ NPs were recorded by using the high resolution scanning electron microscope (HR-SEM). Figure 3a and b shows the HR-SEM images of MCM and CRSGM method prepared CeO_2_ NPs, respectively. The HR-SEM image of CeO_2_ NPs shows the uniform spherical size particles are agglomerate together. Moreover, all the particles are well crystallized and an average grain size is smaller than 100 nm. Furthermore, the nanoparticles were homogeneous and agglomerated with a particle size of sample ***a*** and sample ***b*** is 22.34 and 18.03 nm, respectively^31^. To provide more evidence of CeO_2_ NPs, which was further confirmed by HR-TEM analysis. Figure 4a and b shows the typical HR-TEM image of CeO_2_ NPs prepared by MCM (Sample-***a***) and CRSGM (Sample-***b***) method. The TEM image exhibit that the spherical nanocrystals are uniformly formed and agglomerated together. Moreover, the large size of CeO_2_ NPs obtained from MCM method and smaller size of CeO_2_ NPs obtained from CRSGM method. Besides, the particle size distribution histogram is shown in the inset of Fig. 4a and b. Since, the width of the histogram is narrower and the majority of the particles are in a range 13.29 to 20.35 nm for sample ***a*** and 11.33 to 14.56 nm size for sample ***b***. The surface passivation restricted the crystal growth and prevents aggregation during the synthesis of nanoparticles due to the steric repulsion among the particles^32^,^33^,^34^. Moreover, the mean particle size is very close to the average particle size of XRD pattern particle size.

### Photoluminescence spectroscopy (PL) studies and diffuse reflectance spectroscopy (DRS) studies

**Figure 5:**
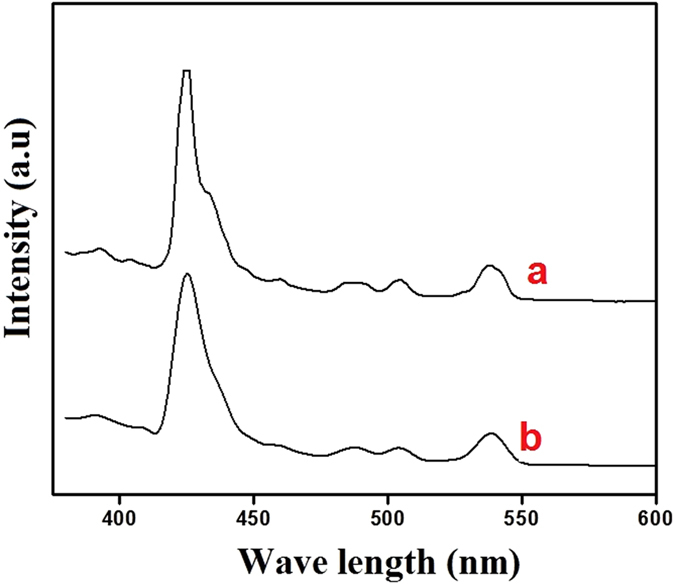
Photoluminescence emission spectra of (**a**) CeO_2_ NPs prepared by the MCM method and (**b**) CeO_2_ NPs prepared by the CRSGM.

Figure 5 shows the photoluminescence spectra (PL) of (a) CeO_2_ NPs prepared by the MCM method and (b) CeO_2_ NPs prepared by the CRSGM, were recorded at room temperature. The PL spectra results show that the synthesized CeO_2_ NPs has the semiconductors properties. Hence, the CeO_2_ NPs display weak excitonic emission in the UV region and strong emission bands in the visible region. Besides, the PL spectrum shows an emission band at around 322 nm, which is attributed to the emission of CeO_2_ NPs. The excitation spectra also reveal a similar degree of enhancement in the intensity. It should correspond to the wide band gap of the CeO_2_ NPs from the conduction band to the valence band^35^,^36^. A strong emission band was observed in the visible region around at 425 nm is due to the presence of defects and two weak emission bands around at 460 and 480 nm may be ascribed to oxygen vacancies. A yellow emission band was observed around 510 and 530 nm, which is attributed to the interstitial oxygen defects and the corresponding energy of this peak. It can also be observed that the intensity of the PL emission gradually increases with increasing the size of CeO_2_ NPs, which may be ascribed to the quantum confinement effect^37^. The crystallite size and emission peaks of CeO_2_ NPs obtained from PL measurements are shown in Table S2. However, the emission peaks were shifted at CRSGM prepared CeO_2_ NPs due to the green reduction. Furthermore, this PL studies is in good agreement with the obtained XRD data.

**Figure 6:**
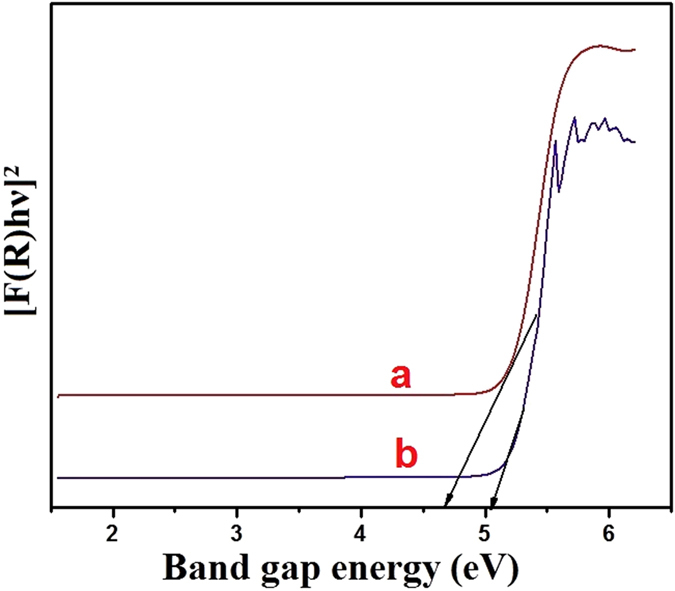
Diffuse reflectance spectra of (**a**) CeO_2_ NPs prepared by the MCM method and (**b**) CeO_2_ NPs sample prepared by the CRSGM.

The optical properties of CeO_2_ NPs were investigated by UV-Visible absorption spectroscopy. The band gap of CeO_2_ NPs can be evaluated from the E_g_ measurements using the Kubelka-Munk (K-M) model and the F(R) is estimated by the F(R) = (1 − R)^2^/2R^38^. Figure 6 shows the plots of [F(R)hυ]^2^ vs band gap energy (eV). The extrapolation of the linear plots until the intersection with hυ axis gives directly the band gap energy value. A blue shift from the bulk band gap value (3.19 eV) is already reported in literature for the CeO_2_ NPs^39^. The obtained band gap values of (a) MCM method prepared CeO_2_ NPs and (b) CRSGM method prepared CeO_2_ NPs is shows at 4.69 and 5.09 eV for particles nanometric dimensions. Therefore, the radiation of CeO_2_ NPs are proposed to be “blue shifted” which reflecting the fact that electrons must fall a greater distance to produce shorter wavelength. The band gap energy exhibits an increasing the particle size due to the decreasing quantum confinement of CeO_2_ NPs. Hence, the higher band gap energy is achieved for the CeO_2_ NPs for the smallest crystallite size^40^. Contradictory to this, many groups have reported a blue shift for the absorbance of nanosized CeO_2_ with proportionate increase in the band gap because of quantum confinement^41^.

Therefore, there are three major counteracting factors determine band gap energy in CeO_2_ NPs such as nanoparticle shape, concentration and quantum confinement effect. Figure 6 shows that the band gap energy exhibits an increasing trend as the crystal size decreases due to the quantum confinement. Hence, the small crystal size of nanoparticles exhibited the large surface area coverage and better adsorption behavior.

### N_2_ adsorption/desorption isotherms (BET)

From the nitrogen adsorption/desorption isotherm (BET), the specific surface areas (S_BET_) together with the pore radius (Rp) and pore volume (Vp) of the CeO_2_ NPs were calculated at 77 K. The MCM (Sample ***a***) and CRSGM (Sample ***b***) method prepared CeO_2_ NPs surface area varied according to the preparation method^42^. The BET surface area of sample ***a*** exhibited at 11.45 m^2^/g and the sample ***b*** exhibited at 19.21 m^2^/g. Furthermore, the pore volume of sample ***a*** & ***b*** is 0.9065 cm^3^/g and 0.9613 cm^3^/g, respectively. The average pore diameter of sample ***b*** is increased to 11.25 Å and sample ***a*** is slightly increased to 10.21 Å. Hence, it is proof that the high surface area of CeO_2_ NPs (sample ***b***) obtained by CRSGM, which is enhance the catalytic activity than that of MCM method synthesis CeO_2_ NPs (sample ***a***).

### Magnetic analysis and electrochemical impedance spectroscopy (EIS) studies

**Figure 7:**
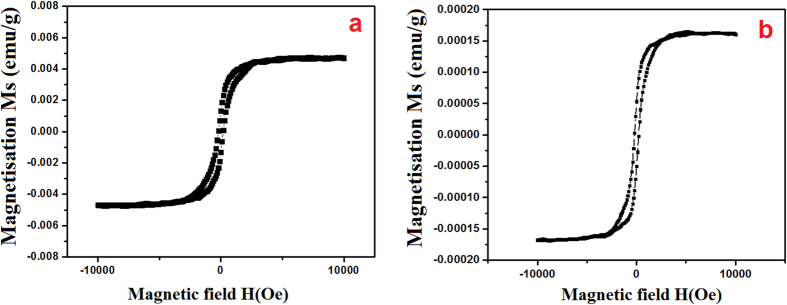
Magnetization curves of (**a**) CeO_2_ NPs prepared by the MCM and (**b**) CeO_2_ NPs prepared by the CRSGM.

The magnetic properties of the samples carried out using a vibrating sample magnetometer in the applied filed range from −10 to +10 kOe at the room temperature. The hysteresis loops (B-H) for the CeO_2_ NPs and the obtained magnetic parameters are shown in Fig. 7. The magnetic properties of MCM prepared CeO_2_ NPs (sample-***a***) is estimated to be 11.63 kOe and 1.872 emu/g. and the magnetic properties of CRSGM prepared CeO_2_ NPs (sample-***b***) is estimated to be 16.31 kOe and 2.561 emu/g, respectively.

Moreover, the saturation magnetization (Ms) of sample ***a*** (20.33 emu/g) is lower than the sample ***b*** (36.67 emu/g) and both the samples are super paramagnetic nature. In addition, the hysteresis curves revealed that the coercivity of the particles, which demonstrates that the CeO_2_ NPs is super paramagnetic. However, the magnetic properties of the samples generally depend on the size, shape, crystallinity, magnetization direction and so on. These corresponding results indicates that the magnetic properties of CeO_2_ NPs is related to the preparation methods^43^. Therefore, it is concluded that well correlation between the magnetic nature and the structural properties of the samples.

**Figure 8:**
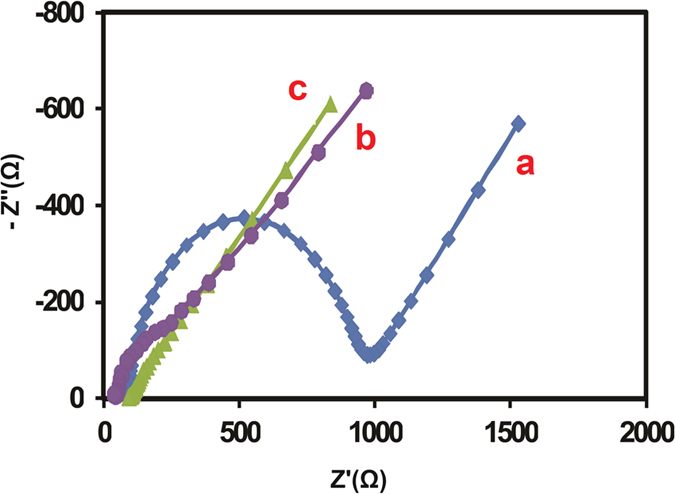
Electrochemical impedance spectra of (**a**) bare GCE, (**b**) MCM prepared CeO_2_ NPs, (**c**) CRSGM prepared CeO_2_ NPs in 0.1 M KCl containing 5 mM [Fe(CN)_6_]^3−/4−^.

On the other hand, the electrochemical impedance spectroscopy (EIS) has been used to identify the charge transfer resistance (*R*_ct_) of the various modified electrodes at electrode/electrolyte interface. Figure 8 shows the EIS plots of (a) bare GCE, (b) MCM prepared CeO_2_ NPs, (c) CRSGM prepared CeO_2_ NPs in 5 mM [Fe(CN)_6_]^3−/4−^ with 0.1 M KCl as a supporting electrolyte. The R_ct_ value of the (c) CRSGM prepared CeO_2_ NPs modified electrode was about 97 Ω, which indicates that the charge transfer resistance was decreased due to the higher electron transfer properties than that of the other modified electrodes.

### Conversion and selectivity studies of benzyl alcohol oxidation

The benzyl alcohol oxidation reaction was carried out under acetonitrile, hydrogen peroxide and CeO_2_ NPs as a catalyst for 6 h at 50 °C. The course of the reaction and the products yield were confirmed by gas chromatography (GC). The catalyst performance exhibited that the based on preparation technique, which is strong influence on both the conversion and product selectivity.

**Figure 9:**
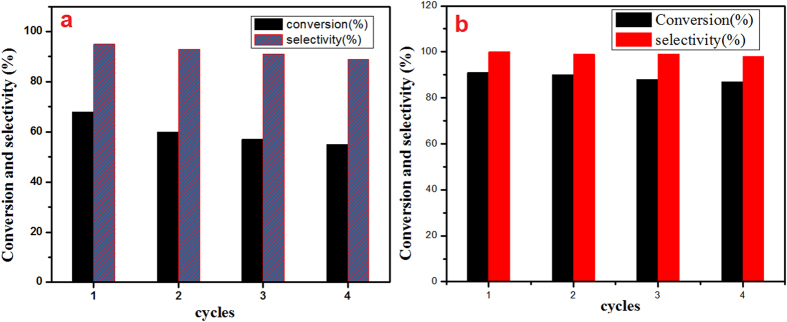
(**a**) Reusability studies of MCM prepared CeO_2_ NPs and (**b**) CRSGM prepared CeO_2_ NPs.

**Figure 10:**
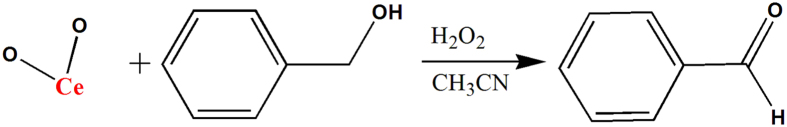
Selective oxidation of benzyl alcohol to benzaldehyde.

Figure 9a and b shows the conversion of benzyl alcohol at the MCM prepared CeO_2_ NPs (sample-***a***) was 68% with 95% selectivity. However, the CRSGM prepared CeO_2_ NPs (sample ***b***) exhibit the conversion was 91% with 100% selectivity. Hence, the results confirmed that the CRSGM prepared CeO_2_ NPs is highly active towards the selective oxidation of benzyl alcohol to benzaldehyde at low temperature (Fig. 10).

It is exhibited the high yield due to the presence of more number of active sites and higher band gap energy. Which is proof that the CRSGM synthesis of CeO_2_ NPs gives better yield with good selectivity than that of the MCM method prepared CeO_2_ NP. Moreover, the MCM method preparation, urea as a fuel which reacts with humid air in atmosphere to produce a toxic gas and damage to health. In contrary, aloe vera plants extract used as a green reducing agent in CRSGM, which is non-polluting, low cost natural materials and an active ingredient in the formation of CeO_2_ NPs^44^.

### Efficiency of the catalyst based on the preparation method

The combustion reaction of CeO_2_ NPs prepared by using urea as a fuel, which is highly exothermic and produced highly propagating flame. While the other samples using with the mixture of fuels were smoldering and burning of a rollup. The reaction samples involving in their higher fuel compositions and portion of atmospheric oxygen would be needed for completion of the reaction^45^. Even though, using Aloe vera plant extract is one of the most accessible, fast, low-energy and soft methods for the synthesis of metal oxide nanomaterials^46^. However, the conventional sol-gel method offers a great advantage in the preparation of CeO_2_ NPs as compared with the microwave combustion method. The formation of a gel with a high degree of homogeneity reduces drastically atomic diffusion during the calcination process therefore, its allowing the formation of desired phases at lower temperature and shorter calcination time reaction. The sol-gel method allows tailoring of the properties of the resulting compounds by the correct choice of the precursors and preparation conditions. As a consequence, the particle size, shape, morphology, crystalline phase, and surface area of CeO_2_ NPs depend on the preparation method. Aloe vera plant extract contains sucrose, maleic, malonic, succinic, tartaric and oxalic acid, which are likely to be responsible for the formation of CeO_2_ NPs^47^. Compared with other methods, the conventional sol-gel route offers the advantages of good control, high homogeneity, producing nanostructure powders as well as low temperature processing. The method was widely used to prepare multicomponent nanomaterial.

### Recapability of the catalyst

For the recapability tests, the reactions were performed under the same reaction conditions as described in catalytic test section. Every time, the catalyst was isolated from the reaction set up at the end of the catalytic reaction, then washed with ethanol and heated at 100 °C. Moreover, the dried catalyst was further reused in a next conversion reaction up to four cycles, which almost shows the same reaction rate as that of the first run (Fig. 9a and b). The slight deactivation of the catalyst may be presumably due to the adsorption of large polar molecules and produce the by-product on the surface of the catalyst. In addition, the adsorption of the polar molecules on the catalyst surface is usually temporary and can be resolved by calcination at high temperature to recover its activity. In this present work, we have proposed the CRSGM synthesis of CeO_2_ NPs catalyst by aloe vera plant extract, which gives the better yield and good selectivity.

### Electrocatalytic oxidation of nitrite at CeO_2_ NPs modified GCE electrode

#### Electrocatalytic oxidation of nitrite at different modified electrodes and effect of different nitrite concentration

**Figure 11:**
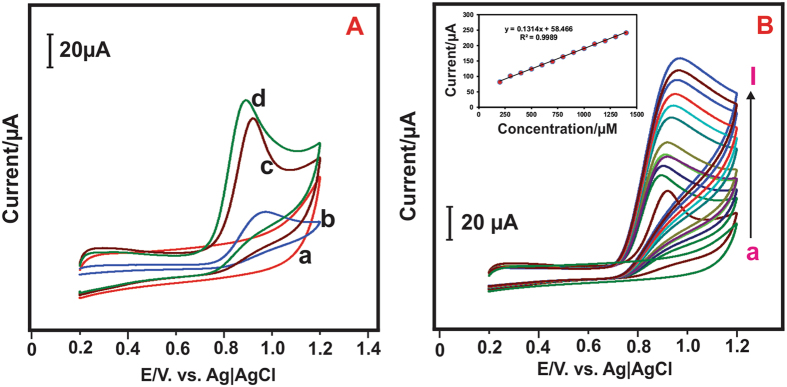
(**A**) Cyclic voltammetry of (a) GCE/CeO_2_ NPs-*b* nanocomposite modified electrode in absence of nitrite and (b) bare GCE, (c) GCE/CeO_2_NPs-*a* (d) GCE/CeO_2_NPs-*b* in 0.05 M PBS (pH 5) containing 200 μM nitrite at scan rate 50 mV s^−1^. (**B**) cyclic voltammetry response of CeO_2_NPs modified electrode at different concentration of nitrite in 0.05 M PBS (pH 5) at the scan rate 50 mV s^−1^.

Figure 11A shows the CV curves of (b) bare GCE, (c) GCE/CeO_2_ NPs-*a* (prepared by the MCM method), (d) GCE/CeO_2_ NPs-*b* (prepared by the CRSGM method) modified electrodes in the presence of 200 μM nitrite in PBS (pH 5) and (a) GCE/CeO_2_NPs- *b* modified electrode shows in the absence of nitrite. As can be seen from the CV studies, the unmodified bare GCE exhibited the nitrite oxidation at the peak potential (*E*_P_) of 0.97 V and the oxidation peak current (*I*_P_) of 32.14 μA. Whereas, the GCE/CeO_2_ NPs-*a* modified electrode shows the oxidized peak potential (*E*_P_) of about 0.92 V and peak current (*I*_P_) of 82.08 μA. However, the GCE/CeO_2_ NPs-*b* modified electrode shows well defined nitrite oxidized peak of *(E*_P_) at 0.82 V and peak current of *I*_P_ at 95.08 μA. There is no noteworthy response for the absence of nitrite at GCE/CeO_2_ NPs-*b* modified electrode. Notably, the oxidation peak current of nitrite was increased at GCE/CeO_2_ NPs-*b* modified electrode than that of GCE/CeO_2_ NPs-*a* modified electrodes. It is also evident from the CV figure, the more active surface area of GCE/CeO_2_ NPs-*b* which plays an important role in the oxidation of nitrite. Therefore, the GCE/CeO_2_ NPs-*b* modified electrode as an excellent electrode material used for the detection of nitrite. Hence, we have been used CRSGM method prepared CeO_2_ NPs (sample-*b*) for throughout full electrochemical experiments. Figure 11B shows the effect of various concentrations of nitrite at GCE/CeO_2_NPs modified electrode in PBS (pH 5) at the scan rate 50 mVs^−1^. The oxidized peak current of nitrite was increased with increasing the concentration of nitrite (100–1000 μM). The Fig. 11B inset shows the linear relation between the peak current (*I*_p_) and concentration of nitrite. Hence, the corresponding linear regression equation is *I*_pa_ = 0.1314x + 58.466 with the correlation coefficient of R^2^ = 0.9989. Which is proof that the CeO_2_NPs modified electrode employed the well electrocatalytic activity towards detection of nitrite.

#### Effect of different pH and different scan rate

**Figure 12:**
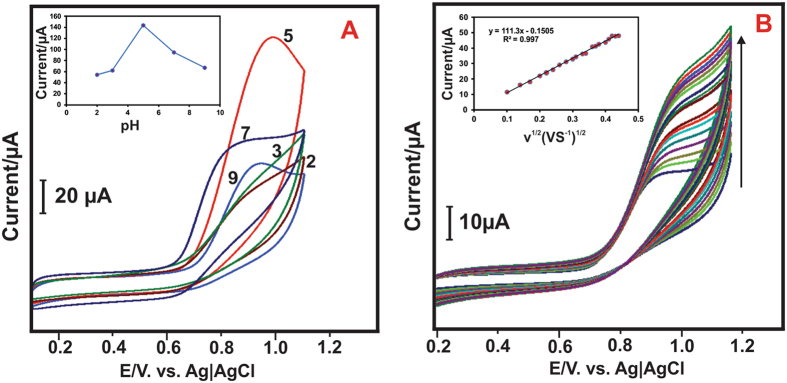
(**A**) Cyclic voltammetry response of CeO_2_NPs modified GCE electrode in 200 μM nitrite solution at different pH (2, 3, 5, 7, 9) at a scan rate of 50 mV s^−1^, (**A**) **inset**, shows the calibration plot for pH vs *I*_P_. (**B**). Cyclic voltammetry response of GCE/CeO_2_NPs in PBS containing 200 μM of nitrite at different scan rates. (**B**) **inset** shows the calibration plot of square roots of scan rate vs. peak current.

Investigation of the effect of pH on the electrochemical response of nitrite oxidation at the modified electrode is more important. The CV experiment was carried out at CeO_2_ NPs modified electrode in various pHs ranging from 2 to 9 in presence of 200 μM nitrite of 50 mVs^−1^. Figure 12A shows the electrocatalytic oxidation peak current of nitrite was increased from pH 2 to 5 and decreasing from pH 6 to 9. This result demonstrate that at lower pH, the NO_2_^−^ ions are unstable because it can be converted to NO and NO_3_^−^. At the same time in higher pHs, the oxidation of nitrite difficult due to the shortage of proton. However, the sensor exhibited higher oxidation performance at pH 5 (Fig. 12A inset). Therefore, we have chosen the pH 5 for all the electrochemical experimental studies. Figure 12B shows the CVs of different scan rate of CeO_2_ NPs modified electrode at PBS (pH 5) in presence of 200 μM nitrite. The results exhibited that the oxidized peak current was increased linearly with increasing the scan rates from 0.01 to 0.17 mVs^−1^. The plot of peak current (*I*_p_) vs. square roots of scan rate (*ν*^1/2^) shows the linear relation and the corresponding linear regression equation is *I*_p_ = 111.3 *ν*^1/2^ (Vs^−1^)^1/2^ + 0.1505 with R^2^ = 0.997 (Fig. 12B inset). Therefore, we concluded that the electro oxidation of nitrite at CeO_2_ NPs modified electrode is diffusion-controlled process.

Moreover, the number of electrons transfer and electron transfer co-efficient of this reaction can be estimated by the following equations


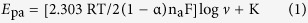



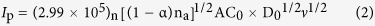


Where K is a constant, n_a_ is 1 and substituting the slope value in the equation 1, the value of α is 0.52 for the nitrite oxidation. There withal, the number of electrons involved in the nitrite oxidation can estimated by the equation 2. The nitrite oxidation at the CeO_2_ NPs modified electrode for irreversible process controlled by diffusion reaction. For equation (2), the A is the electrode area, D_0_ is the diffusion coefficient of nitrite and C_0_ is the concentration of nitrite. By substituting all the values in equation 2, the value of n calculated to be 2, this is good accordance with previous research article. The overall nitrite oxidation can be expressed by the following equation (3)^48^,^49^


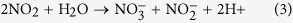


Hence, the electrocatalytic oxidation of nitrite at the CeO_2_ NPs modified electrode as a two electrons transfer reaction.

#### Amperometric determination of nitrite at CeO_2_ NPs modified electrode

**Figure 13:**
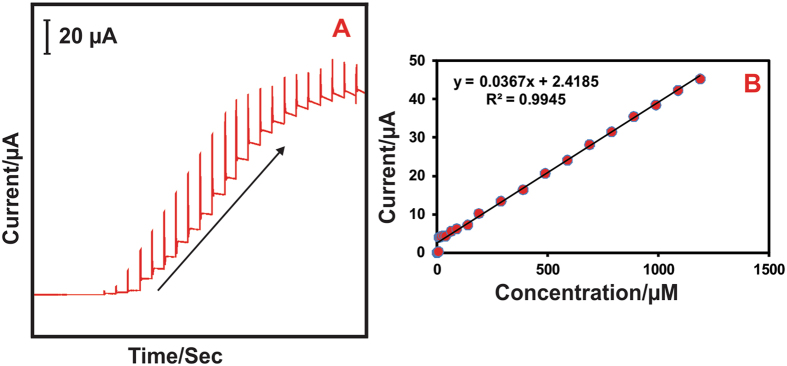
(**A**) Amperometric response for the different concentration of nitrite in PBS (pH 5), E_app_ = 0.8 V. (**B**) The calibration plot of peak current vs. nitrite concentration.

Figure 13A exhibited the typical amperometric response of nitrite oxidation at CeO_2_ NPs modified rotating disc electrode (RDE) at the rotation speed of 2000 rpm in PBS (pH 5). The successive addition of different concentration of nitrite into PBS with 50 s interval and the applied potential (*E*_app_) of + 0.8 V. It can be seen that a well-defined stable amperometric response was appeared for the every addition of nitrite; the concentration of nitrite was increased up to 1200 μM. As shown in Fig. 13B, the oxidation peak current of nitrite was linearly increased with increasing the concentration of nitrite (0.02–1200 μM). The corresponding linear regression equation was expressed as I_p_/μA = 0.0367x + 2.4185, R^2^ = 0.9945. Moreover, the limit of detection (LOD) and sensitive was calculated to be 0.21 μM and 1.7238 μAμM^−1^ cm^−2^ respectively. However, the CeO_2_ NPs modified electrode has exhibited wide linear range, low detection limit and long linear range than that of other nitrite sensor. These obtained analytical results are compared with some other nitrite sensors and shown in Table 1. Moreover, the CeO_2_ NPs modified electrode has the well stability, repeatability and reproducibility properties (S.3). Therefore, the CeO_2_ NPs modified electrode has been used for the determination of nitrite in different real samples.

#### Interference and real sample analysis of CeO_2_ NPs/GCE fabricated electrode

**Figure 14:**
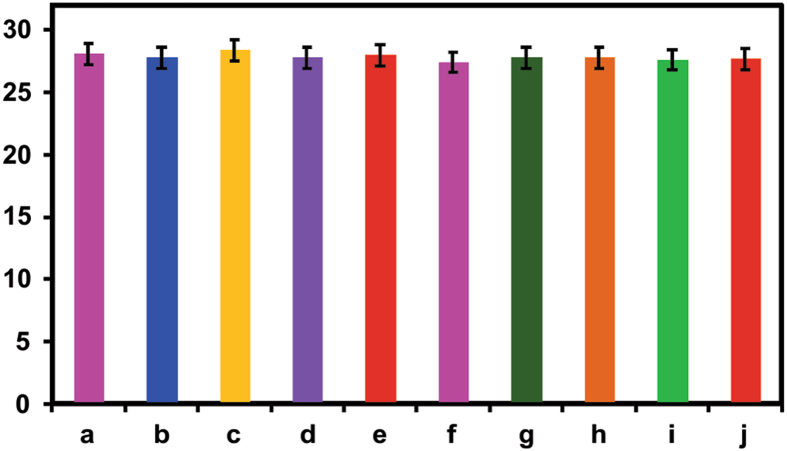
Relative error of the interference studies towards detection of (**a**) nitrite (50 μM) in presence of higher concentration of other interfering compounds such us ((**b**) K^+^, (**c**) Ca^+^, (**d**) Zn^+^, (**e**) Cr^+^, (**f**) Sr^+^, (**g**) 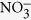, (**h**) I^−^, (**i**) Fructose, (**j**) Ascorbic acid even in the presence of other interfering compounds.

The selectivity of CeO_2_NPs modified electrode was investigated towards the detection of nitrite in the presence of common interference ions and biological molecules. Figure 14 shows the amperometric response of CeO_2_ NPs modified electrode, the well amperometric response was observed for the 50 μM additions of nitrite (a) and there is no notable response was appeared for additions of other interfering ions. Hence, this interference study exhibited that the CeO_2_ NPs modified electrode selectively detect the nitrite.

Besides, the practical feasibility of the CeO_2_NPs modified sensor has been demonstrated by amperometric method in various water samples. Before performing the real sample analysis, the collected water sample was filtered and removes the solid content. The standard addition method was used for the real sample analysis and the recoveries are 94.3%, 103% and 102% is shown in Table 2. From this result demonstrates that the modified sensor has acceptable recoveries in real sample analysis. Therefore, the proposed modified electrode has been used for the determination of nitrite in different real sample analysis.

## Conclusions

The CeO_2_ NPs were prepared by both MCM and CRSGM methods. The effect of the preparation, structural morphology, optical, magnetic and catalytic activity for the selective oxidation of benzyl alcohol was investigated. The CeO_2_ NPs have been successfully synthesized by a simple and rapid microwave-assisted combustion method using urea as the fuel. The successful formation of CeO_2_ NPs was confirmed by HR-SEM, HR-TEM and XRD. The synthesized CeO_2_NPs showed good optoelectronic properties. Moreover, the magnetic properties of CeO_2_NPs exhibit the super paramagnetic behavior at room temperature. Thus, highly effective CRSGM route is achieved for the selective oxidation of benzyl alcohol to benzaldehyde. Moreover, the CeO_2_NPs modified electrode exhibited an enhanced electro catalytic activity towards the detection of nitrite. Moreover, the modified electrode shows the excellent analytical response such as low-level detection (0.21 μM), long linear range response (0.02–1200 μM), sensitivity (1.7238 μAμM^−1^ cm^−2^), acceptable selectivity, repeatability and reproducibility. Moreover, the modified electrode was applied for the determination of nitrite in different water samples with satisfactory recovery.

## Experimental Details

### Materials and Methods

All the chemicals and reagents used were of analytical grade without further purification. Cerium nitrate (Ce(NO_3_)_3_.6H_2_O), sodium nitrite (NaNO_2_) and urea (CO(NH_2_)_2_) were purchased from sigma Aldrich. Millipore water was used all the experimental condition. The aloe vera plant leaves were collected from the local agricultural fields in Chennai, India and used for the nanomaterial’s preparation. The phosphate (0.05 M) buffer solution (PBS) was prepared by using Na_2_HPO_4_ and NaH_2_PO_4_, the pH was adjusted by H_2_SO_4_ or NaOH. Prior to each electrochemical experiment, the electrolyte were purged with purified N_2_ for 15 min.

### Preparation of CeO_2_ nanoparticles and GCE/CeO_2_ NPs modified electrode

Cerium nitrate (0.62 g) and urea (0.60 g) were dissolved separately in millipore water and mixed together in a glass beaker at room temperature for about 1 h to obtain a homogeneous solution. The homogeneous solution was poured into a silica crucible and placed inside the microwave oven for irradiation. Further, the microwave was irradiated over the solution for 7 min at 750 watts out-put power and frequency of 2.45 GHz. Then, the solution boils and undergoes dehydration followed by decomposition with the evolution of gases. After that, the solution reaches the spontaneous combustion and the solution becomes a solid. The obtained CeO_2_ NPs was washed well with alcohol and dried were labeled as a **sample-*****a*** (prepared by microwave combustion method). Moreover, the cerium nitrate (0.62 g) and *aloe vera* plant. extract were dissolved in de-ionized water and under constant stirring for 5 hr at room temperature until a clear transparent solution was obtained. The obtained solution was dried in an air oven at 120 °C for 5 h. The powders were then sintered at 400 °C at a heating rate of 5 °C/min for 3 h in muffle furnace and the obtained CeO_2_ NPs was labeled as a **sample-*****b*** (prepared by conventional route sol-gel method). The prepared CeO_2_ NPs (10 mg) (**sample** ***a*** **&** ***b***) was dispersed in DMF (1 mL) and drop casted on the GCE surface then dried in room temperature and used for the electrochemical detection of nitrite.

### Characterization of CeO_2_ nanoparticles

The structural studies of nanomaterials were carried out by using a Philips X’ pert diffractometer for 2 h values ranging from 10° to 80° using Cu Kα radiation at λ = 0.154 nm. The SEM studies and the energy dispersive X-ray analysis (EDX) of CeO_2_ NPs have been performed by JEOL-JSM6 360 high-resolution scanning electron microscope (HR-SEM). The transmission electron microscope studies were carried out by Philips-TEM (CM20). The diffuse reflectance UV–visible spectra were recorded using Cary 100 UV-Visible spectrophotometer. The optical properties of nanomaterials were recorded using Varian Cary Eclipse fluorescence spectrophotometer. The magnetic properties were investigated by using a PMC Micro Mag 3900 model vibrating sample magnetometer (VSM). The N_2_ adsorption-desorption isotherms (BET) of the samples were measured by using an automatic adsorption instrument (Quanta chrome Quadra win gas absorption analyzer). The electrochemical measurement was carried out using CHI410 and CHI 750a electrochemical workstation (Shanghai Chen Hua. Co).

### Catalytic test

The selective oxidation of benzyl alcohol was carried out in batch reactor, which operated under atmospheric conditions. Moreover, the oxidant of H_2_O_2_ (5 mmol) was added along with 0.3 g of the CeO_2_ NPs (**sample** ***a*** & **sample** ***b***) and the whole reaction contents were heated at 50 °C in acetonitrile medium for 6 h. Besides, the reaction content contained with the three necked round bottom flask equipped with a reflux condenser and thermometer. After the catalytic reaction, the oxidized product was collected and studied by using Agilent GC spectrometer. The GC was used as a DB wax column (capillary column) of length 30 mm and helium was used as the carrier gas. In addition, the GC technique was used to know the conversion percentage of the obtained product. Furthermore, the obtained catalytic oxidation products of alcohols were confirmed by Tollen’s and Fehling’s tests.

## Additional Information

**How to cite this article**: Tamizhdurai, P. *et al*. Environmentally friendly synthesis of CeO_2_ nanoparticles for the catalytic oxidation of benzyl alcohol to benzaldehyde and selective detection of nitrite. *Sci. Rep.* **7**, 46372; doi: 10.1038/srep46372 (2017).

**Publisher's note:** Springer Nature remains neutral with regard to jurisdictional claims in published maps and institutional affiliations.

## Supplementary Material

Supplementary Information

## Figures and Tables

**Figure 1:**
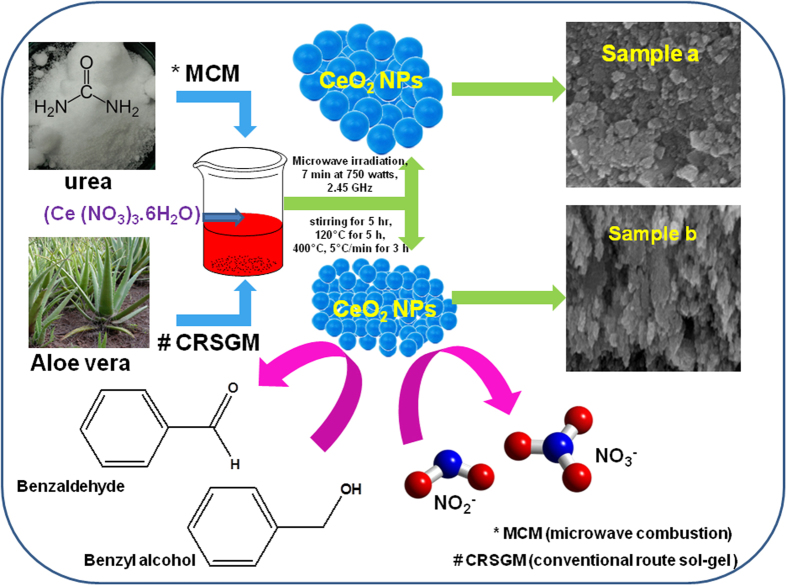
Schematic diagram for the synthesis of cerium oxide nanoparticle (CeO_2_ NPs) by the microwave combustion method (MCM) and conventional route sol-gel method (CRSGM).

**Table 1:** Comparison of the analytical performance of GCE/CeO_2_ NPs modified electrode with other nitrite sensors.

Electrode materials	Linear range (μM)	Limit of detection (μM)	References
GCE/^*a*^CeO_2_ NPs	0.02–1200	0.21	This work
^*b*^RGO-^*c*^MWCNT-^*d*^Pt/^*e*^Mb/^*f*^GCE	1–1200	0.93	50
^*g*^Au@Fe_3_O_4_/^*h*^Cys/GCE	3.6–1000	0.82	24
Fe_3_O_4_@Pt nanoparticles	0.33–1300	0.109	51
GR-^*i*^CS/AuNPs/GCE	1–380	0.25	52
PtNPs	10–1000	5	53
^*j*^CuO/GCE	1.0–91.5	0.36	54
^*k*^GO–Ag nanocomposite	10–180	2.1	55
^*l*^B-doped cubic ^*m*^SiCNWs	5–8000	0.5	56
Nano-Au/^*n*^P3MT/GCE	10–1000	2.3	57
Fe_3_O_4_/rGO	1–92	0.3	58
^*o*^G_4_-NH_4_/MWCNT	5–50	2	59

Abbreviation: ^***a***^Cerium oxide nanoparticles, ^***b***^Reduced graphene oxide, ^***c***^Multiwalled carbon nanotube, ^***d***^Platinum, ^***e***^myoglobin, ^***f***^Glassy carbon electrode, ^***g***^Gold, ^***h***^L-Cysteine, ^***i***^Copper oxide, ^***j***^chitosan, ^***k***^graphene oxide, ^***l***^silver nanocomposite, ^***m***^boran, ^***n***^silicon carbide (SiC) nanowires, ^***o***^Amine-terminated poly(amidoamine).

**Table 2:** Determination of nitrite in real sample using CeO_2_ NPs modified electrode.

Samples	Added (μM)	Found (μM)	Recovery (%)	*RSD (%)
River water	10	9.43	94.3	3.5
Tap water	10	10.3	103	3.3
Sea water	10	10.2	102	3.2

*Relative standard deviation of 3 individual measurements.

## References

[b1] Bekyarova E., Fornasiero P., Kaspar J. & Graziani M. CO oxidation on Pd/CeO_2_-ZrO_2_ catalysts. Catal. Today. 45, 179–183 (1998).

[b2] Bumajdad A., Eastoe J. & Mathew A. Cerium oxide nanoparticles prepared in self-assembled systems. Adv. Colloid Interface Sci. 147, 56–66 (2009). 19027889 10.1016/j.cis.2008.10.004

[b3] Slostowski C., Marrea S., Bassat J. M. & Aymonier C. Synthesis of cerium oxide-based nanostructures in near- and supercritical fluids. J. Supercrit. Fluids. 84, 89–97 (2013).

[b4] Sun C. W., Li H., Zhang H. R., Wang Z. X. & Chen L. Q. Controlled synthesis of CeO_2_ nanorods by a solvothermal method. Nanotechnology. 16, 1454–1463 (2005).

[b5] Campbell C. T. & Peden C. H. F. Chemistry: oxygen vacancies and catalysis on ceria surfaces. Science. 309, 713–714 (2005). 16051777 10.1126/science.1113955

[b6] Zhang F. . Cerium oxide nanoparticles: size-selective formation and structure analysis. Appl. Phys. Lett. 80, 127–129, (2002).

[b7] Hu J., Li Y., Zhou X. & Cai M. Preparation and characterization of ceria nanoparticles using crystalline hydrate cerium propionate as precursor. Mater. Lett. 61, 4989–4992 (2007).

[b8] Wang H. . Preparation of nano crystalline ceria particles by sonochemical and microwave assisted heating methods. Phys. Chem. Chem. Phys. 4, 3794–3799 (2002).

[b9] Czerwinski F. & Szpunar J. A. The nano crystalline ceria sol-Gel coatings for high temperature applications. J. Sol-Gel Sci. Technol. 9, 103–114 (1997).

[b10] Yao S. Y. & Xie Z. H. Deagglomeration treatment in the synthesis of doped-ceria nanoparticles via co precipitation route. J. Mater. Process. Technol. 186, 54–59 (2007).

[b11] Subramanian V., Burke W., Zhu H. & Wei B. J. Novel Microwave Synthesis of Nano crystalline SnO_2_ and Its Electrochemical Properties. Phys. Chem. C. 112, 4550–4556 (2008).

[b12] Srivastava A., Lakshmikumar S. T., Srivastava A. K. & Jain K. Gas sensing properties of nano crystalline SnO_2_ prepared in solvent media using a microwave assisted technique. Sens. Actuators, B. 126, 583–587 (2007).

[b13] Koseoglu Y., Baykal A., Gozuak F. & Kavas H. Structural and magnetic properties of CoxZn_1−x_Fe_2_O_4_ nanocrystals synthesized by microwave method. Polyhedron. 28, 2887–2892 (2009).

[b14] Sertkol M., Koseoglu Y., Baykal A., Kavas H. & Toprak M. S. Synthesis and magnetic characterization of Zn 0.7, Ni 0.3, Fe_2_O_4_ nanoparticles via microwave-assisted combustion route. J. Magn. Magn. Mater. 322, 866–871 (2010).

[b15] Laokul P., Amornkitbamrung V., Seraphin S. & Maensiri S. Characterization and magnetic properties of nanocrystalline CuFe_2_O_4_, NiFe_2_O_4_, ZnFe_2_O_4_ powders prepared by the Aloe vera extract solution. Curr. Appl. Phys. 11, 101–108 (2011).

[b16] Boudreau M. D. & Beland F. A. An evaluation of the biological and toxicological properties of Aloe barbadensis (miller) Aloe vera. J. Environ. Sci. Health Part C. 24, 103–154 (2006). 10.1080/10590500600614303 16690538

[b17] Maensiri S. . Indium oxide (In_2_O_3_) nanoparticles using Aloe vera plant extract: Synthesis and optical properties J. Optoelectron. Adv. Mater. 10, 161–165 (2008).

[b18] Ragupathi C., Vijaya J. J., Kennedy L. J. & Bououdina M. Combustion synthesis, structure, magnetic and optical properties of cobalt aluminate spinel nano crystals. Ceram. Int. 40, 13067–13074 (2014).

[b19] Sakthinathan S. . A non-covalent interaction of Schiff base copper alanine complex with green synthesized reduced graphene oxide for highly selective electrochemical detection of nitrite. RSC Adv. 6, 107416–107425 (2016).

[b20] Cui, L. . Controlled chitosan coated Prussian blue nanoparticles with the mixture of graphene nanosheets and carbon nanoshperes as a redox mediator for the electrochemical oxidation of nitrite. Sens. Actuators, B. 161, 641–647 (2012).

[b21] Zhao K. . Determination of nitrite with the electrocatalytic property to the oxidation of nitrite on thionine modified aligned carbon nanotubes. Electrochem. Commun. 9, 65–70 (2007).

[b22] Zhang Y., Zhao Y., Yuan S., Wang H. & He C. Electrocatalysis and detection of nitrite on a reduced graphene/Pd nanocomposite modified glassy carbon electrode. Sens. Actuators, B. 185, 602–607 (2013).

[b23] Salome J. P. . Electrochemical assay of the nitrate and nitrite reductase activities of Rhizobium japonicum. Biosens. Bioelectron. 24, 3487–3491 (2009). 19482466 10.1016/j.bios.2009.04.044

[b24] Yu C., Guo J. & Gu H. Electrocatalytic oxidation of nitrite and its determination based on Au@Fe3O4 nanoparticles. Electroanalysis. 22, 1005–1011 (2010).

[b25] Ma X. . Electrochemical detection of nitrite based on glassy carbon electrode modified with gold-polyaniline-graphene nano composites. RSC Adv. 4, 57842–57849 (2014).

[b26] Davis J., Moorcroft M. J., Wilkins S. J., Compton R. G. & Cardosi M. F. Electrochemical detection of nitrate and nitrite at a copper modified electrode. Analyst. 125, 737–742 (2000).

[b27] Rahim A. . Electrochemical detection of nitrite in meat and water samples using a mesoporous carbon ceramic SiO_2_/C electrode modified with *in situ* generated manganese (II) phthalocyanine. Electroanalysis. 26, 541–547 (2014).

[b28] Zhang L. & Yi M. Electrochemical nitrite biosensor based on the immobilization of hemoglobin on an electrode modified by multiwall carbon nanotubes and positively charged gold nanoparticle. Bioprocess Biosyst Eng. 32, 485–492 (2009). 18941796 10.1007/s00449-008-0268-7

[b29] Becheri A., Durr M., Nostro P. L. & Baglioni P. Synthesis and characterization of zinc oxide nanoparticles: application to textiles as UV-absorbers. J. Nanopart. Res. 10, 679–689 (2008).

[b30] Samiee S. & Goharshadi E. K. Effects of different precursors on size and optical properties of ceria nanoparticles prepared by microwave-assisted method. Mater. Res. Bull. 47, 1089–1095 (2012).

[b31] Bandyopadhyay S., Paul G. K., Roy R., Sen S. K. & Sen S. Study of structural and electrical properties of grain-boundary modified ZnO films prepared by sol–gel techniqu eMater. Chem. Phys 74, 83–9 (2002).

[b32] Lee S. Y. & Harris M. T. Surface modification of magnetic nanoparticles capped by oleic acids: Characterization and colloidal stability in polar solvents. J. Colloid Interf. Sci. 293, 401–408 (2006). 10.1016/j.jcis.2005.06.062 16054635

[b33] Tjong S. C. & Chen H. Nano crystalline materials and coatings. Mater. Sci. Eng. R. 45, 1–88 (2004).

[b34] Jain N., Wang Y. J., Jones S. K., Hawkett B. S. & Warr G. G. Optimized steric stabilization of aqueous ferro fluids and magnetic nanoparticles. Langmuir. 26, 4465–4472 (2009). 10.1021/la903513v 19950943

[b35] Roychowdhury A., Pati S. P., Mishra A. K., Kumar S. & Das D. Magnetically addressable fluorescent Fe_3_O_4_/ZnO nano composites: Structural, optical and magnetization studies. J. Phys. Chem. Solids. 74, 811–818 (2013).

[b36] Zhuang H., Wang J., Liu H., Li J. & Xu P. Structural and optical properties of ZnO nanowires doped with magnesium. Acta Phys. Pol. A 119, 819–823 (2011).

[b37] Yilmaz S., Bacaksiz E., Mcglynn E., Polat I. & Ozcan S. Structural, optical and magnetic properties of Zn_1−x_MnxO micro-rod arrays synthesized by spray pyrolysis method. Thin Solid Films. 520, 5172–5178 (2012).

[b38] Ramaiah K. S., Pilkington R. D., Hill A. E., Tomlinson R. D. & Bhatnagar A. K. Structural and optical investigations on CdS thin films grown by chemical bath technique. Mater. Chem. Phys. 68, 22–3 (2001).

[b39] Miao J. J., Wang H., Lia Y. R., Zhub J. M. & Zhua J. J. Ultrasonic-induced synthesis of CeO_2_ nanotubes. J. Cryst. Growth. 281, 525–529 (2005).

[b40] Li L. S., Hu J. T., Yang W. & Alivisatos, A. P. Band gap variation of size-and shape-controlled colloidal CdSe quantum rods. Nano Letters. 1, 349–351(2001).

[b41] Maensiri S. . Egg white synthesis and photoluminescence of platelike clusters of CeO_2_ nanoparticles. Cryst. Growth Des. 7, 950–955 (2007).

[b42] Hirst S. M. . Anti-inflammatory Properties of Cerium Oxide Nanoparticles. small. 24, 2848–2856 (2009). 10.1002/smll.200901048 19802857

[b43] Wei Y.. Synthesis of Fe_3_O_4_ Nanoparticles and their Magnetic Properties. Procedia Eng. 27, 632–637 (2012).

[b44] Ragupathi C., Vijaya J. J., Surendhar P. & Kennedy L. J. Comparative investigation of nickel aluminate (NiAl_2_O_4_) nano and microstructures for the structural, optical and catalytic properties. Polyhedron. 72, 1–7 (2014).

[b45] Tahmasebi K. & Paydar M. H. The effect of starch addition on solution combustion synthesis of Al_2_ O_3_–ZrO_2_ nanocomposite powder using urea as fuel. Mater. Chem. Phys. 109, 156–163 (2008).

[b46] Ragupathi C., Vijaya J. J. & Kennedy L. J. A new approach: synthesis, characterization and optical studies of nano zinc aluminate. Adv. Powder Technol. 25, 267–273 (2014).

[b47] Ragupathi C., Vijaya J. J., Kumar R. T. & Kennedy L. J. Elective liquid phase oxidation of benzyl alcohol catalyzed by copper aluminate nanostructures. J. Mol. Struct. 1079, 182–188 (2015).

[b48] Lin C. Y., Balamurugan A., Lai Y. H. & Ho K. C. A novel poly (3, 4 ethylene dioxy thiophene)/iron phthalo cyanine/multi-wall carbon nanotubes nanocomposite with high electrocatalytic activity for nitrite oxidation. Talanta. 82, 1905–191 (2010). 20875594 10.1016/j.talanta.2010.08.010

[b49] Guidelli G., Pergola F. & Raspi G. Voltammetric behavior of nitrite ion on platinum in neutral and weakly acidic media. Anal. Chem. 44, 745–749 (1972). 22309533 10.1021/ac60312a018

[b50] Mani V., Dinesh B., Chen S. M. & Saraswathi R. Direct electrochemistry of myoglobin at reduced graphene oxide-multiwalled carbon nanotubes-platinum nanoparticles nanocomposite and biosensing towards hydrogen peroxide and nitrite. Biosens. Bioelectron. 53, 420–427 (2014). 24211453 10.1016/j.bios.2013.09.075

[b51] Ma M., Xie J., Zhang Y., Chen Z. & Gu N. Fe_3_O_4_@Pt nanoparticles with enhanced peroxidase-like catalytic activity. Mater. Lett. 105, 36–39 (2013).

[b52] Xue W. . Simultaneous electrochemical determination of sulphite and nitrite by a gold nanoparticle/graphene-chitosan modified electrode. Chinese J Anal Chem. 41, 1232–1237 (2013).

[b53] Miao P., Shen M., Ning L., Chen G. & Yin Y. Functionalization of platinum nano p articles for electrochemical detection of nitrite. Anal Bioanal Chem. 399, 2407–2411 (2011). 21225247 10.1007/s00216-010-4642-3

[b54] Zhang L., Yuan F., Zhang X. & Yang L. Facile synthesis of flower like copper oxide and their application to hydrogen peroxide and nitrite sensing. Chem Cent J. 5, 75 (2011). 22133166 10.1186/1752-153X-5-75 PMC3245445

[b55] Ikhsan N. I. . Facile synthesis of graphene oxide-silver nanocomposite and its modified electrode for enhanced electrochemical detection of nitrite ions. Talanta. 144, 908–914 (2015). 26452907 10.1016/j.talanta.2015.07.050

[b56] Yang T., Zhang L., Hou X., Chen J. & Chou K. C. Bare and boron-doped cubic silicon carbide nanowires for electrochemical detection of nitrite sensitively, Sci Rep 6, 24872, doi: 10.1038/srep24872 (2016). 27109361 PMC4843007

[b57] Huang X., Li Y., Chen Y. & Wang L. Electrochemical determination of nitrite and iodate by use of gold nanoparticles/poly (3-methylthiophene) composites coated glassy carbon electrode. Sensor. Actuat. B. 134, 780–786 (2008).

[b58] Teymourian H., Salimi A. & Khezrian S. Fe_3_O_4_ magnetic nanoparticles/reduced graphene oxide nanosheets as a novel electrochemical and bioeletrochemical sensing platform. Biosens. Bioelectron. 49, 1–8 (2013). 23708810 10.1016/j.bios.2013.04.034

[b59] Zhu N., Xu Q., Li S. & Gao H. Electrochemical determination of nitrite based on poly (amidoamine) dendrimer-modified carbon nanotubes for nitrite oxidation. Electrochem. Commun. 11, 2308–2311 (2009).

