# Supplementary material for: Environmentally friendly synthesis of CeO2 nanoparticles for the catalytic oxidation of benzyl alcohol to benzaldehyde and selective detection of nitrite
Source: Sci Rep. 2017 Apr 13;7:46372. doi: 10.1038/srep46372 (PMC5390321; doi:10.1038/srep46372)
Supplement: Supplementary Information [file srep46372-s1.doc]

**Supporting information**

**Environmentally friendly synthesis of CeO_2_ nanoparticles for the catalytic oxidation of benzyl alcohol to benzaldehyde and selective detection of nitrite**

P. Tamizhdurai^1, 3^, Subramanian Sakthinathan ^2^, Shen-Ming Chen ^2^*, R. Shanthi^1^*, S. Sivasanker^3^, P.Sangeetha ^4^

^1^Department of Chemistry, Anna University, Chennai 600025, India

^2^Electroanalysis and Bioelectrochemistry Lab, Department of Chemical Engineering and Biotechnology, National Taipei University of Technology, No. 1, Section 3, Chung-Hsiao East Road, Taipei 106, Taiwan, ROC

^3^National Centre for Catalysis Research, Indian Institute of Technology, Chennai, India.

^4^Department of Chemistry, School of Advanced Sciences, VIT University, Vellore.

**Corresponding authors**

^1*^K.Shanthi, shanthiramesh@annauniv.edu

^2*^S.M. Chen, [smchen78@ms15.hinet.net](mailto:smchen78@ms15.hinet.net), Fax: +886 2270 25238; Tel: +886 2270 17147,

**S. Table 1 Crystallographic data for CeO_2_ NPs**

| Sample | Crystal structure | unitcell volume (Å^3^) | Lattice parameter  (A˚) | Crystal size (nm) |
| --- | --- | --- | --- | --- |
| JCPDS | cubic phase | 157.790 | 5.31 | **---** |
| Sample a | cubic phase | 157.772 | 5.67 | 23.83 |
| Sample b | cubic phase | 157.710 | 5.98 | 18.23 |

**S. Table.2. Crystallite size, emission peaks and region of cerium oxide calculated by PL measurements**

| Samples | Crystallite size (nm) | Emission peaks (nm) | Region (nm) |
| --- | --- | --- | --- |
| Sample a | 23.83 | 425,464,483,510 and532 | Violet- green-yellow |
| Sample b | 18.23 | 421,461,480,506 and528 | Violet- green-yellow |

**S3. Stability, repeatability and reproducibility studies of CeO_2_ NPs/GCE** **modified electrode**

The stability of the CeO_2_ NPs modified RDE electrode was investigated by the amperometric technique in the presence of 100 µM nitrite for 2500 s. The experimental condition is similar in section 5.3. It can be seen that the oxidized peak current response lost only 2.2 % from the initial response current. Hence, the results reveal that the good operational stability of the modified electrode. Moreover, the repeatability and reproducibility of the modified electrode was carried out by the CV studies in the presence of 100 µM nitrite in PBS (pH 5). The sensor exhibited the RSD of about 3.25 % for the 5 repetitive measurements carried out by the single modified electrode. However, the modified electrode shows the appreciable reproducibility of 3.16 % for the 5 measurements carried out by the 5 different modified electrodes. Therefore, the obtained results indicate that the CeO_2_ NPs modified electrode have good repeatability and reproducibility behavior.
